# Potential of Silver Nanoparticles in Imaging Diagnostics and Image-Guided Applications: A Narrative Review

**DOI:** 10.3390/ph19050722

**Published:** 2026-05-01

**Authors:** Vera Gledacheva, Stoyanka Nikolova

**Affiliations:** 1 Department of Medical Physics and Biophysics, Faculty of Pharmacy, Medical University of Plovdiv, 4002 Plovdiv, Bulgaria; 2 Department of Organic Chemistry, Faculty of Chemistry, University of Plovdiv, 4000 Plovdiv, Bulgaria

**Keywords:** silver nanoparticles, synthesis, green synthesis, image-guided therapy

## Abstract

**Background/Objectives**: Silver nanoparticles (AgNPs) are highly valuable nanomaterials due to their unique optical and physicochemical properties. AgNPs have a lot of promise as contrast-enhancing and diagnostic agents in image-guided treatment. With a focus on their incorporation into image-guided and theranostic approaches, this narrative review attempts to assess the current function of AgNPs in imaging diagnostics. **Methods**: Using major scientific databases, such as PubMed, Web of Science, and Scopus, a narrative literature review has been conducted with an emphasis on recent preclinical and experimental research examining AgNP-based systems for diagnostic imaging applications. The design of the NPs, surface functionalization, imaging modality, and diagnostic performance of the evaluated studies were analyzed. **Results**: Due to their surface plasmon resonance and tunable physicochemical properties, AgNPs show great promise in a variety of imaging techniques, such as optical imaging, computed tomography (CT), and multimodal platforms, according to the reviewed literature. Functionalized AgNPs emerged as agents in image-guided therapy due to their improved target selectivity, enhanced imaging contrast, and signal amplification in tissues. **Conclusions**: AgNPs are appealing nanoscale platforms for image-guided methods and imaging diagnostics. Despite their encouraging preclinical results, some key issues, such as toxicity, biocompatibility, and clinical translation, remain critical. AgNP-based therapeutic and diagnostic systems will need to overcome these constraints in the future.

## 1. Introduction

Medical imaging is an essential part of modern diagnostics that enables early disease detection, treatment planning, and therapy monitoring [1,2]. Contrast agents are mostly employed in traditional imaging methods. To increase sensitivity and specificity, they are most frequently utilized in nuclear imaging, computed tomography (CT), magnetic resonance imaging (MRI), and optical imaging [3,4,5]. Advanced diagnostic platforms have been necessary due to the short circulation times, low target specificity, and possible toxicity of many therapeutically employed contrast agents. Nanotechnology created new techniques with multifunctional capabilities and nanoscale imaging agents. In addition to their strong signal-generating capabilities and suitability for a range of imaging methods, metallic nanoparticles (NPs) have attracted increased interest recently [6,7,8].

Among the various metallic nanoparticles, gold NPs and iron oxide NPs are also widely investigated for imaging applications. Gold NPs are particularly valued for their high plasmonic stability and tunable optical properties, which make them suitable for optical and CT imaging [9,10]. Iron oxide NPs from the other side are extensively used as contrast agents in MRI due to their superparamagnetic behavior [11,12]. Recently, precious metals, such as Au, Ag, and Pt, were applied in magnetic nanomaterials synthesis. The obtained magneto-optical materials exhibit exceptional synergistic plasmonic and magnetic capabilities [13].

Nevertheless, the present review is specifically focused on AgNPs, owing to their unique combination of optical, physicochemical, and biological properties.

Among the other NPs, AgNPs have been shown to absorb electromagnetic radiation in the visible spectrum between 380 and 450 nm, which makes them ideal candidates for optical imaging and signal amplification [14,15,16]. Moreover, AgNPs’ potential use as contrast agents for CT imaging is supported by the improved X-ray absorption, due to their large atomic number [17,18].

Cancer treatment is one of the most difficult problems in medicine. The most popular cancer treatments are chemotherapy and radiation. However, due to their long-term and ongoing negative effects, they are regarded as invasive techniques [19]. Bacterial resistance to antibiotics, which has proven difficult to treat infections, particularly in a hospital setting, is another growing worry [20]. As a result, treatments with application of light’s local incidence, such as photothermal therapy and photodynamic therapy, have become viable, minimally invasive options. These treatments have shown a number of benefits, including little drug resistance, fewer adverse effects, and non-invasiveness [19,20].

In order to create light-absorbing agents that can enhance the reduction–oxidation reactions at the targeted site or convert light into thermal energy with low invasiveness and high efficiency, the design of sophisticated nanomaterials for phototherapy has expanded dramatically in recent years [21]. A number of NPs, such as metallic nanostructures, semiconductor quantum dots, carbon dots, and upconversion NPs, have been developed to get around the drawbacks of conventional photosensitizers [22,23]. Among these nanomaterials, metallic nanostructures, including AgNPs and gold (Au-), show promise for use in phototherapy [23,24,25]. These materials are appealing as photosensitizing agents because of their nanoscale localized surface plasmon resonance (LSPR), a phenomenon that greatly influences their thermal, optical, and electrical properties [24,25].

AgNPs can transform light energy into thermal energy and function as an antenna to increase photosensitizers’ efficiency [26]. Additionally, the enhanced permeability and retention (EPR) effect, which permits higher penetration and longer retention durations in cancer cells compared to normal cells, is exploited by the use of NPs in biomedical applications [27,28]. Additionally, AgNPs’ surface can be altered to increase selective bioaccumulation and prolong their lifetime in the systemic circulation [29]. These characteristics can improve the accumulation of AgNPs in the intended location and boost the targeting of sick cells, enabling localized therapy [30].

Additionally, the biochemical modification and functionalization of the NPs’ surface are frequently required to increase their stability in biological fluids and decrease toxicity. For example, AgNP-embedded nanoshell structures are employed in photothermal therapy and cancer imaging to locate cancer cells by absorbing light and eliminating them through the photothermal effect [18].

AgNPs are increasingly being used therapeutically, particularly in phototherapy techniques. AgNPs’ exceptional performance as phototherapeutic agents, specifically for photodynamic therapy (PDT), photothermal therapy (PTT), and photodynamic inactivation of microorganisms, has been attributed to their plasmonic characteristics [31].

**Figure 1:**
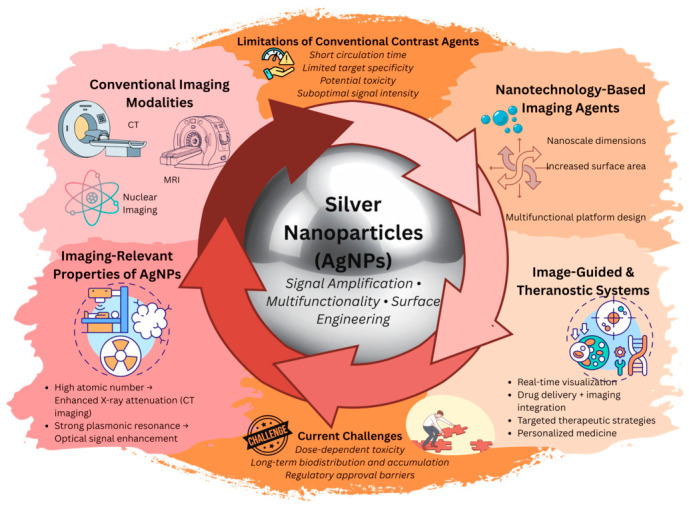
Conceptual framework of AgNP-based platforms for imaging diagnostics and image-guided applications. This image was created with Canva (https://www.canva.com/bg_bg/).

AgNPs have been investigated as image-guided and theranostic systems with diagnostic and therapeutic functions on a single nanoscale platform (Figure 1) [32,33,34,35]. These methods offer real-time observation of the dispersion of NPs and therapeutic response, which may enable more precise and tailored medical treatments. It is also worth mentioning that the AgNP-based treatment of human cell cultures may induce cytotoxicity, inflammatory responses in a cell-type-dependent manner, and genotoxicity [36]. Particular attention is paid to their use in optical, CT, and multimodal imaging. A CT scan with high contrast makes it simple to diagnose blood clots, fractures, malignant tumors, infections, internal injuries and bleeding, and cardiovascular diseases. They also help with biopsies, postoperative status monitoring, and determining the exact surgical area. Although iodine-based contrast agents have historically been the preferred option, radiologists are now also interested in high-atomic-number NPs, such as gold, tantalum, bismuth, silver, etc. [37].

Therefore, the primary objective of this narrative review is to provide an up-to-date overview and critical evaluation of developments in the use of AgNP-based image diagnostics.

## 2. Materials and Methods

The PRISMA 2020 guidelines for literature screening and reporting were followed in the conduct of this review. To find relevant studies, a systematic literature search was conducted in PubMed, Web of Science, and Scopus. Combinations of keywords such as “silver nanoparticles”, “AgNPs”, “image diagnostics”, “contrast agents”, “computed tomography”, “image-guided therapy”, and “theranostics” were used in the search method. Only English-language peer-reviewed articles were included in the search.

Studies were included if they applied AgNP-based systems for diagnostic imaging, used preclinical or experimental data that have been reported, and provided enough methodological information about the synthesis of NPs and their imaging capabilities. Studies focused on the therapeutic benefits of AgNPs without imaging components or focused on silver compounds or silver ions were not included. Editorial papers and conference abstracts have been excluded, as well. The size and shape of the NPs, the synthesis method, the surface functionalization, the imaging modality, the signal enhancement mechanism, the experimental model, and the published diagnostic results were examined from each study.

**Figure 2:**
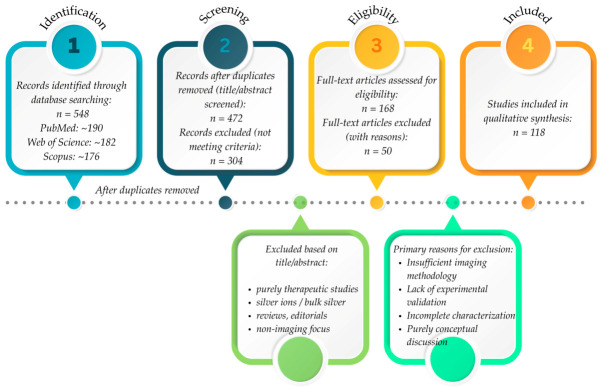
PRISMA 2020 flow diagram illustrating the literature search and study selection process for AgNP-based imaging diagnostics. This image was created with Canva (https://www.canva.com/bg_bg/).

A PRISMA flow diagram is used to summarize the research selection procedure (Figure 2).

## 3. Results and Discussion

The application of AgNPs for diagnostic imaging has drawn more attention within the last ten years. Preclinical experimental models, such as in vitro cell-based systems and small animal models, constitute the early translational stage of AgNP-based imaging platforms and are the subject of most studies [38,39,40].

### 3.1. AgNPs in Optical Imaging Diagnostics

The most established use of AgNPs is in optical imaging diagnostics [41] due to their strong localized surface plasmon resonance (LSPR). The collective oscillation of conduction band electrons in response to incoming light causes AgNPs to display LSPR. The LSPR is produced at the AgNPs’ surface when they are exposed to an electromagnetic field because the electrons in the metals’ conduction band fluctuate collectively and in resonance with the light frequency. AgNPs’ optical characteristics are greatly affected by this phenomenon, which also modifies how they interact with their surroundings. Because the electrons in NPs smaller than the wavelength of light move coherently against the external electric field, the surface plasmon oscillation creates a dipole moment [23,42].

By modifying their size and form, these nanostructures’ LSPR may be adjusted, which also affects the hue of the colloidal suspension. Colloidal suspensions of spherical AgNPs have a yellow hue and a single absorption band at about 400 nm, which is indicative of a distinct dipole oscillation mode of the conduction electrons. Conversely, asymmetrical nanostructures, like prismatic AgNPs, show several plasmon resonance modes with a maximum at 550–1000 nm, leading to colloidal suspensions with colors spanning from violet to blue and a wider absorption band [43,44]. AgNPs can therefore provide more efficient absorption and increased local heating for treatments that employ visible to near-infrared light [31].

**Figure 3:**
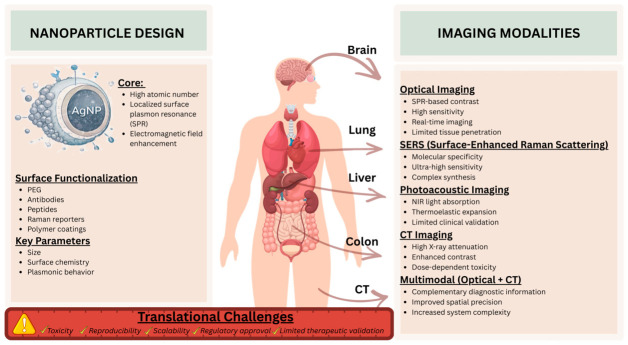
AgNP-Based Image-Guided Applications. This image was created with Canva (https://www.canva.com/bg_bg/).

SPR enables efficient light absorption and scattering, making them appropriate for fluorescence-based imaging [45,46], surface-enhanced Raman scattering (SERS) [47,48], and photoacoustic imaging [41,49,50] (Figure 3).

Depending on their size, the AgNPs display significantly enhanced localized SPR bands in the UV-Vis wavelengths. When the Ag concentration increases, the SPR bands are significantly stronger, and when the Ag sublimation occurs, they gradually weaken. Additionally, as NP size decreases, the Vis area of SPR bands is easily blue-shifted.

SERS-based techniques showed remarkable analytical sensitivity across the examined modalities, while photoacoustic imaging enhances the diagnostic potential of AgNPs by converting absorbed optical energy into acoustic signals, thereby increasing imaging depth and spatial resolution compared to purely optical methods. Aggregation of colloidal NPs is still favored in situations demanding exceptionally high sensitivity among the many techniques presently available for creating SERS substrates. In fact, due to their high concentration in hot spots, aggregation of AgNPs has been identified as the cause of single-molecule detection in SERS [51,52].

**Figure 4:**
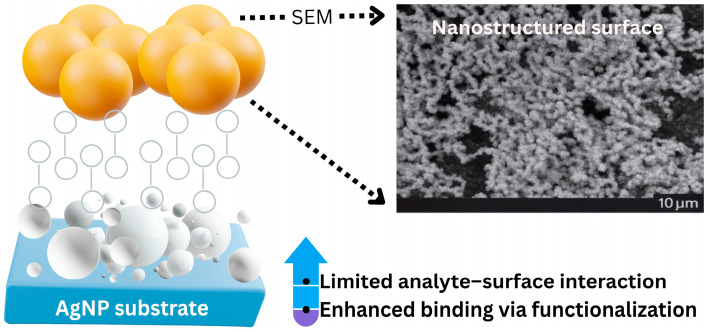
Schematic representation of the sensor (**left**). Scanning electron microscopy image of AgNP aggregates on the sensor surface (**right**). This image was created with Canva (https://www.canva.com/bg_bg/).

Raman discrimination cannot distinguish between closely related species in complicated samples, such as peptides that differ only slightly in sequence, even though SERS is a selective approach because it generates a vibrational fingerprint of the analyte. Additionally, the analyte molecule must have affinity for the metal surface in order to reach the SERS substrate surface, which is a prerequisite for obtaining SERS activity. Conversely, regardless of how active the substrate is, the analyte of interest will not exhibit the SERS effect if it has no affinity for the surface at all. By covering the surface of the NPs with a material that either directly binds to the target molecule or encourages adsorption, these disadvantages have been addressed (Figure 4). For instance, these surface-coated SERS sensors have enabled quantitative in vivo glucose monitoring [53] and the detection of compounds like polycyclic aromatic hydrocarbons that have no affinity for uncoated surfaces [54].

A unique and quick technique for the qualitative detection of protein kinase activity is another intriguing example of these modified SERS substrates. AgNPs were placed on a glass substrate and covalently bonded to a target to create the sensor kinase-recognition peptide via a thiol group in the peptide’s amine terminus. The phosphate group’s covalent attachment to the peptide tyrosine residue following the kinase activity caused a noticeable shift in the Raman spectra, showing a collapse of the 848/828 cm^−1^ doublet to a single peak at about 830 cm^−1^. Even in crude cell lysates, the described approach demonstrated selectivity [55].

Apart from the SERS active substrates, the fiber-optic SERS sensor is another appealing sensor configuration. The substrate serves as both an excitation and a detection mechanism in this kind of sensor. For instance, biological samples, such as plant tissues or microbiological cells, could be recorded with high spatial resolution thanks to glass fiber tips coated with AgNPs. This sensor’s significantly lower laser power requirement prevented sample photoinduced destruction [56]. In a different instance, water considerably below the ppb detection limit included crystal violet molecules, a pollutant used in aquaculture [57].

SERS tags are a new SERS sensor architecture that has become a potent tool in biological tests in recent years. In a nutshell, a SERS tag is a tagging agent that is chemically encoded. A SERS reporter molecule, or a molecule with a high Raman cross-section, adsorbed on the surface of an AgNP—which may or may not be further encased in a protective shell—forms the fundamental structure of the SERS tag. After the fundamental structure is put together, it could be coupled to an antibody or other recognition element. The SERS reporter molecule’s Raman signature will indicate whether the labeling agent is present during a detection procedure.

Due to their advantages over conventional fluorescence labels, including narrower emission peaks, more optical signatures, high-level multiplexing, single laser excitation for multiple label detection, and higher stability, SERS tags are a great substitute for fluorescence-based encoding techniques.

As previously stated, a SERS tag can be produced by merely adsorbing a SERS reporter molecule on the surface of a NP. This straightforward yet efficient method has been used, for instance, to create a sandwich-type immunosensor to identify the hepatitis B surface antigen [58]. Table 1 summarizes representative experimental investigations and their salient features.

Organic dyes are among the most widely used fluorescence probes due to their low cost and small molecular size [77]. However, organic dyes have low hydrophilicity and photostability and are intrinsically hazardous when used as contrast agents for near-infrared imaging [78]. They also have inadequate stability in biological systems and show low quantum yields with limited detection sensitivity [45]. To increase imaging efficiency, these dyes have been chemically coupled with biological macromolecules or NPs [79]. Numerous NPs, such as quantum dots, noble metal clusters, including AgNPs, and carbon-based nanomaterials (carbon dots, graphene, nanodiamonds, etc.), have been produced and extensively researched in biological imaging to get around the drawbacks of organic dyes and fluorescent proteins [80]. AgNPs can concentrate selectively in the tissues by functionalizing with dyes, which enhances imaging contrast [33,34,81,82,83,84,85,86]. Despite the excellent diagnostic efficacy of these optical imaging techniques, their clinical translation is limited due to the tissue penetration, inconsistent signal repeatability, and biosafety. Table 2 presents the adaptability and the existing limits of AgNP-based optical imaging modalities.

### 3.2. CT Imaging

A diagnostic imaging test with improved spatial and density resolution is called CT [114]. It creates a series of X-ray images using ionizing radiation. The photographs are processed using computational methods to provide detailed, high-quality images. High-quality 3D images of internal organs, the spine, the spinal column, and soft tissues, including blood and blood vessels, can be produced [115]. Cardiovascular disease, musculoskeletal disorders, infectious diseases, cancer and malignancies, lung diseases, chest problems, appendicitis, and internal organ damage are among the conditions for diagnosis [116,117,118,119]. Currently, iodine- and bromine-sulfate-based contrast agents have received clinical approval. Iodine is often administered intravenously into the spinal canal and other body cavities [120,121]. The patient’s health and medical factors are taken into consideration when choosing the contrast agent and delivery method. In addition to serving as therapeutic agents for non-conventional cancer therapy, a number of NPs can serve as contrast agents. Because of their multimodality, these can be referred to as theragnostic agents. This lowers toxicity and improves efficacy by lowering the dosage of NPs needed for medicinal and diagnostic purposes. AgNPs have the potential to replace conventional iodine-based CT contrast agents [18,122]. The significant X-ray properties result from silver’s high atomic number.

NPs are perfect for in vivo tomography and a target-specific approach because they have a longer circulation lifetime, superior contrast densities, and an easily functionalized surface compared to iodinated compounds [123,124]. A clear 3D image of the area under investigation can be produced by using NPs as contrast agents in CT scans [125]. The targeted contrast agents composed of inorganic NPs improve CT imaging contrast by several orders of magnitude, even at low X-ray dosages [126]. This reduces the amount of time that people are exposed to highly ionizing X-rays and the risks that come with them.

The literature data showed that administered AgNPs have longer circulation duration and improve CT contrast, compared to other small-molecule treatments [37,127]. The size and surface of AgNP-based CT contrast agents enhance imaging performance and biodistribution. These two characteristics may contribute to more trustworthy imaging outcomes [17,127]. Additionally, targeted AgNPs preferentially accumulate in tissues, enabling localized contrast amplification [31,37,128]. CT-based applications, however, give rise to worries about long-term silver buildup and dose-dependent toxicity. Compared to gold NPs, which have been studied more thoroughly for CT imaging, AgNPs’ in vivo clearance and long-term exposure implications are still not well studied [129,130].

AgNP-based systems are useful when therapeutic planning is helped by visualization in image-guided applications. Combining the characteristics of optical imaging with CT or other imaging techniques, AgNPs enable complementary diagnostic information [40,131]. AgNP-based image-guided application allowed visualization of the target location and NPs distribution by combining therapeutic planning, procedural guiding, and diagnostic imaging capabilities (Figure 3). Table 3 summarizes how AgNPs have been investigated in a variety of image-guided approaches, such as CT-guided diagnostics, optical imaging-assisted therapies, and multimodal platforms intended to improve diagnostic accuracy and spatial precision.

Important characteristics of NPs, including size, surface functionalization, and plasmonic behavior, are important in determining imaging contrast, targeting effectiveness, and image-guided workflow compatibility. Although these techniques demonstrate a promising diagnostic support in preclinical models, most research continues to focus on preliminary imaging rather than verified therapy outcomes.

### 3.3. Biocompatibility and Toxicity

AgNPs have significant diagnostic advantages, but their usage in clinical imaging applications is restricted by a number of safety and biocompatibility concerns. The AgNP toxicity is significantly influenced by particle size, surface coating, and given dose [139,140]. Smaller AgNPs usually exhibit increased cellular absorption and greater cytotoxicity [141,142].

Gliga et al. found that only 10 nm particles were cytotoxic, regardless of surface coating [143]. On the other hand, the comet assay revealed that all AgNPs examined increased total DNA damage after 24 h, indicating separate mechanisms for cytotoxicity and DNA damage. Nevertheless, intracellular reactive oxygen species generation was not observed. By examining particle aggregation in cell media, cellular absorption, intracellular localization, and Ag release, the authors investigated the reasons for NP’s increased toxicity. Surface functionalization methods, like drug-loading, polymer coatings, and biomolecule conjugation, have been shown to improve biocompatibility and decrease toxicity [144,145,146,147].

From a translational perspective, regulatory uncertainty and a lack of long-term in vivo proof remain major challenges.

The size of AgNPs has long been a topic of interest. Particle size is a basic property that affects the surface effect of nanomaterials. At the same dose, different sizes even have different effects. Specifically, the toxicity of small-sized AgNPs is much higher than that of large particle size [148]. One of the reasons could be biological transport. AgNPs with larger diameters may enter/exit cells through ion channels, while AgNPs with small diameters can directly pass through the cell membrane and act in the cells. On the other hand, a small particle size has a larger specific surface area, which can better play the role of AgNPs. Therefore, in this section, we have reviewed the toxicity of AgNPs of different diameters.

To find the toxicity of different-sized AgNPs, in vivo tests on mice with oral administration for six weeks were applied [149]. The result indicated that the brain, liver, kidney, and other organs have small-diameter AgNPs (from 22 to 71 nm). Bigger-sized AgNPs (up to 323 nm) were not detected in those tissues.

Furthermore, due to excessive Ag^+^ release, reactive oxygen species (ROS) production, and possible harm to DNA, lipids, and cellular proteins, increasing AgNP dosage reduces cell viability [150,151]. However, the toxicity of cysteine-stabilized AgNPs, both positively charged (AgNPs-Cys(+)) and negatively charged (AgNPs-Cys(−)), was examined by Oćwieja et al. [152]. According to the findings, positively charged AgNPs are more harmful than their negatively charged counterparts, lowering lymphocyte viability and producing more noticeable genotoxicity and membrane disruption. Vuković et al. [153] compared the cytotoxic effects of AgNPs with three different surface charges: positive (ε-poly-L-lysine, PLL), neutral (polyvinylpyrrolidone, PVP), and negative (bis(2-ethylhexyl) sulfosuccinate sodium, AOT). According to this study, positively charged AgNPs have a more noticeable cytotoxic effect.

Recordati et al. [154] investigated the effects of oral exposure to 0.25 and 1 mg Ag.kg^−1^ for four weeks in mice. This was accomplished using spherical AgNPs with a diameter of 10 nm that were stabilized with citrate. The mice given Ag salts had greater ratios of Ag accumulation, and the scientists noted that the accumulation of AgNPs in organs was dosage-dependent. Furthermore, a considerable amount of Ag was still found in the brain and testis 28 days after treatment, even after a period of recuperation. In a different investigation, mice were exposed to AgNPs-PVP (with 5 and 50 nm) for 28 days at doses of 1 and 3 mg Ag.kg^−1^ [155]. Regardless of AgNP size, these authors found that Ag was present in every organ examined. The majority of the Ag^+^ was eliminated through the urinary system; animals treated with Ag salt (AgNO_3_) and AgNPs-polyvinylpyrrolidone with 50 nm showed the greatest levels, whereas mice administered with AgNO_3_ and AgNPs with 5 nm had the highest concentrations of Ag^+^ in their feces. Additionally, these authors demonstrated that the effects of AgNPs were size-dependent, with the smaller AgNPs exhibiting a widespread accumulation in many organs, including the liver, kidney, spleen, and lung. In light of these findings, AgNP toxicity must be reduced by a strategic design that carefully takes dosage, surface charge, and nanoparticle size into account, guaranteeing a balance between safety and therapeutic efficacy.

AgNPs’ ability to produce ROS, which can damage cellular membrane integrity and initiate apoptotic pathways, is largely responsible for their cytotoxic effects. When molecular oxygen and other reactants are present, AgNPs easily oxidize, releasing Ag^+^ ions. The cell experiences increased oxidative stress as a result of this process, which also increases the generation of ROS. Furthermore, a crucial cellular signaling and defense mechanism that is intimately linked to oxidative stress is endoplasmic reticulum (ER) stress. Its major function is to preserve protein homeostasis by avoiding the aggregation of unfolded or misfolded proteins and minimizing their accumulation. On the other hand, exposure to AgNPs may cause severe ER stress by disrupting ER homeostasis, impairing appropriate protein folding, and encouraging the buildup of aberrant proteins within the ER lumen. AgNPs may also affect mitochondrial function through ROS-independent mechanisms, according to experimental data. These findings suggest that three main mechanisms—oxidative stress, ER stress, and mitochondrial dysfunction mediated by non-ROS pathways—are essential to AgNP-induced toxicity [149].

Rosário et al. [155] showed that these nanoparticles’ impacts varied with size, suggesting that they may be linked to distinct health risks. Although the effects of AgNP exposure may be attributed to both the release of Ag^+^ ions and the nanoparticulate form, ion dissolution appeared to be a significant factor in evaluating the kinetics and effects of AgNPs. Up to the final day of recovery (28 days), smaller particles appeared to have long-lasting inflammatory effects with a substantial neutrophil influx. Particulate silver appeared to be the cause, as these effects resembled those of the AgNO_3_ exposure. In mice, 5 nm AgNPs were widely dispersed and accumulated for at least 28 days in particular organs like the liver, kidney, spleen, and lung [155].

The immune system may have reacted differently to larger particles by recruiting eosinophils, which are larger phagocytic cells. Additionally, 50 nm AgNPs showed a good clearance at 28 dpi and a distribution profile comparable to AgNO_3_. Finally, the size once again dictated the excretion of AgNPs. Although feces also contained a significant concentration of AgNP5, the urine appeared to be the primary route for excretion.

AgNPs can also translocate into the systemic circulation after passing through physiological barriers, which would put secondary organs under oxidative stress. While the cellular redox state in the liver may be linked to a stress response and biliary excretion of silver as an Ag-GSH complex, the rise in the GSSG:GSH ratio and GSH content may have been a reaction to the cell antioxidant requirements in the lung. It was demonstrated that modeling the ADME of AgNPs in seven distinct mouse tissues was effective for the liver and heart.

AgNPs are more thoroughly examined than other metallic NPs because of their possible environmental impact and recognized antibacterial activity [36,156,157].

**Figure 5:**
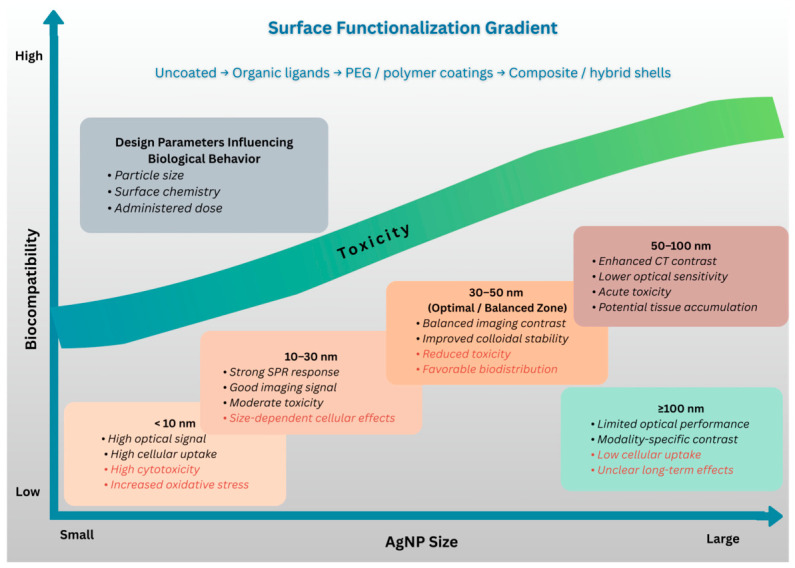
Conceptual Framework of AgNP Size–Toxicity–Imaging Relationships. This image was created with Canva (https://www.canva.com/bg_bg/).

AgNPs’ biological activity and imaging capabilities are strongly influenced by NP size and surface coating, as well as by control over biodistribution, cellular uptake, signal intensity, and toxicity profiles (Figure 5). Ag-NPs are effective for bioimaging, especially in cancer treatment, because they are also good at penetrating cells [158,159,160,161,162,163]. Ag-NPs’ surface functionalization enables targeted bioimaging, which offers insightful information on molecular interactions and cellular functions.

Table 4 demonstrates how smaller AgNPs often exhibit better imaging signals due to their stronger plasmonic effects. They also correlate with higher cytotoxicity and nonspecific tissue accumulation. Conversely, surface modification methods, such as drug loading, polymer coatings, and biomolecule conjugation, improve colloidal stability, reduce aggregation, and decrease biological side effects.

Overall, AgNPs have a great deal of promise as diagnostic imaging agents, especially in optical and CT-based techniques. Improved imaging contrast and tailored diagnostic performance are possible due to their special plasmonic capabilities, customizable physicochemical features, and ability to be functionalized. In the majority of the research, the lack of consistent evaluation has been identified as a significant limitation. Due to variations in NPs production, surface modification, dose methods, and imaging methodologies, direct comparisons of studies are challenging. Future studies will be more concentrated on performing methodical, head-to-head comparisons between AgNP-based contrast agents and clinically validated imaging agents in order to accurately describe their relative advantages and disadvantages. For the translational development of AgNP in imaging technologies, long-term safety and biodistribution remain significant challenges. Despite the extensive reporting of short-term imaging performance, thorough assessments of long-term exposure, NP clearance, and potential Ag accumulation are usually absent. Standardized toxicity and biosafety evaluation techniques that use both in vitro and in vivo models must be developed in order to support clinical relevance and regulatory review.

Finally, addressing these methodological, safety, and translational challenges will be crucial to fully understand the diagnostic potential of AgNP-based imaging platforms and to facilitate their progression from experimental systems to clinically meaningful tools.

## 4. Conclusions

In general, the current study highlights recent advancements in the utilization of AgNPs as diagnostic imaging agents. AgNPs’ beneficial plasmonic and physicochemical properties enable enhanced imaging contrast, signal amplification, and targeted diagnostics. AgNPs are appealing choices for theranostic and image-guided methods in biological research because of these qualities. The practical application of AgNP-based imaging systems is still hampered by problems with biocompatibility, long-term safety, and the absence of standardized evaluation protocols, despite encouraging preclinical results. The already published studies highlight the need for comprehensive toxicity assessment, methodical comparison studies, and the development of therapeutically applicable imaging techniques. In conclusion, research on AgNP-based imaging systems is a rapidly expanding area of nanomedicine. Continued multidisciplinary efforts integrating nanotechnology, imaging science, and regulatory considerations will be essential to bring these platforms closer to safe and effective diagnostic applications in precision medicine.

## Figures and Tables

**Table 1:** Overview of representative studies on AgNP-based imaging diagnostics.

Application Focus	Experimental Model	AgNP Size (nm)	Surface Modification	Imaging Modality	Key Outcome	References
Tumor imaging	In vitro	20–40	PEGylation	Optical imaging	Enhanced contrast and prolonged circulation	[59,60]
Inflammation imaging	In vivo	10–30	Peptide conjugation	Optical imaging	Targeted signal accumulation	[61,62]
Vascular imaging	In vivo	30–60	Polymer coating	CT imaging	Improved vessel contrast	[63,64]
Multimodal diagnostics	In vitro/in vivo	40–80	Hybrid shell	Optical + CT	Complementary imaging information	[5,65]
Bacterial infection imaging	In vitro/in vivo	10–25	Antibiotic conjugation	Optical imaging	Selective binding to bacterial cells and enhanced detection	[66]
Brain imaging (BBB targeting)	In vivo	15–35	Transferrin functionalization	Optical imaging	Improved blood–brain barrier penetration	[67,68]
Lymph node mapping	In vivo	20–50	PEG + dye labeling	Optical imaging	Efficient lymphatic drainage and node visualization	[69,70]
Theranostics	In vitro/in vivo	30–70	Drug-loaded polymer shell	Optical + photoacoustic	Simultaneous imaging and therapeutic delivery	[33,71]
Renal clearance studies	In vivo	5–15	Citrate coating	Optical imaging	Rapid clearance and kidney tracking	[72,73]
Gastrointestinal imaging	In vivo	50–100	Biocompatible polymer coating	CT imaging	Enhanced contrast in GI tract	[32,74,75,76]

**Table 2:** AgNP-based optical imaging approaches and diagnostic performance.

Optical Imaging Technique	AgNP Feature Enabling Imaging	Functionalization Strategy	Diagnostic Advantage	Limitation	References
Fluorescence enhancement	Plasmon-enhanced emission	Antibody conjugation	Improved sensitivity and localization	Signal quenching at high concentrations	[87,88,89]
Light scattering imaging	Strong plasmonic scattering	Polymer stabilization	Real-time visualization	Limited depth penetration	[90]
SERS	Electromagnetic field amplification	Raman reporters; targeting ligands	Ultra-high sensitivity	Complex probe design	[91,92]
Photoacoustic imaging	Efficient photothermal conversion	PEGylation; hybrid structures	Improved tissue penetration	Thermal safety considerations	[93,94]
Metal-enhanced fluorescence	LSPR coupling	Dye conjugation; silica shell	Increased fluorescence intensity and photostability	Distance-dependent effects	[95,96,97,98]
Dark-field microscopy	Strong elastic scattering (LSPR)	Antibody or peptide targeting	High-contrast single-particle imaging	Limited tissue penetration	[99,100,101]
Hyperspectral imaging	Tunable plasmonic spectra	Surface coating with targeting ligands	Spectral fingerprint-based detection	Complex data analysis	[102,103,104]
Fluorescence lifetime imaging	Plasmon-modified decay rates	Fluorophore coupling	Quantitative imaging independent of intensity	Instrumentation complexity	[105,106]
Two-photon imaging	Nonlinear optical response enhancement	Polymer or ligand functionalization	Deeper tissue imaging and reduced photodamage	Lower efficiency compared to dyes	[107]
Photothermal imaging	Heat generation upon absorption of light	PEGylation; antibody targeting	High sensitivity for single particles	Thermal effects on tissue	[108,109,110,111]
Upconversion-assisted imaging	Energy transfer with upconversion nanoparticles	Hybrid AgNP–UCNP systems	Reduced background autofluorescence	Complex nanostructure synthesis	[112,113]

**Table 3:** Image-guided therapies and key features of AgNPs in diagnostics.

Imaging Modality	AgNP Properties	Functionalization	Diagnostic Advantages	Limitations	References
Optical imaging	Localized SPR, light scattering	Antibodies, peptides, polymers	High sensitivity; signal amplification; real-time imaging	Limited tissue penetration depth; photothermal effects	[132]
Surface-enhanced Raman scattering (SERS)	Strong electromagnetic field enhancement	Raman reporters; targeting ligands	Ultra-high sensitivity; molecular specificity	Complex synthesis; limited in vivo validation	[133,134]
Photoacoustic imaging	Efficient light absorption, heat generation	Polymer coating; hybrid nanostructures	Improved imaging, high contrast	Potential thermal effects; limited clinical studies	[135,136]
CT	High atomic number; X-ray attenuation	PEG; targeting ligands	Prolonged circulation time; enhanced contrast	Dose-dependent toxicity; accumulation concerns	[17,137]
optical/CT	Optical, X-ray contrast properties	Multifunctional surface coatings	Complementary diagnostics; image-guided therapies	Increased system complexity; regulatory challenges	[60,138]

**Table 4:** AgNP surface coating and size effects on imaging performance and toxicity.

AgNP Size Range	Surface Coating	Imaging Performance	Toxicity	References
<10 nm	Uncoated, not stabilized	High optical signal; cellular uptake	Higher cytotoxicity; increased oxidative stress	[164,165]
10–30 nm	small organic ligands	Strong SPR response; good signal	Moderate toxicity; size-dependent cellular effects	[166,167]
30–50 nm	PEG or polymer coatings	Balanced imaging contrast; improved stability	Reduced toxicity; improved biocompatibility	[168,169,170,171]
50–100 nm	PEG, protein coatings	Lower optical sensitivity; enhanced CT contrast	acute toxicity; potential accumulation	[172,173]
100 nm or >100 nm	Composite or hybrid shells	Limited optical performance; modality-specific	Low cellular uptake; unclear and long-term effects	[174,175,176,177,178]

## Data Availability

No new data were created or analyzed in this study.
